# Long-term Bowel Dysfunction and Decline in Quality of Life Following Surgery for Colon Cancer: Call for Personalized Screening and Treatment

**DOI:** 10.1097/DCR.0000000000002377

**Published:** 2022-08-19

**Authors:** Sanne J. Verkuijl, Edgar J.B. Furnée, Wendy Kelder, Christiaan Hoff, Daniel A. Hess, Fennie Wit, Ronald J. Zijlstra, Monika Trzpis, Paul M.A. Broens

**Affiliations:** 1 Department of Surgery, Anorectal Physiology Laboratory, University of Groningen, University Medical Center Groningen, Groningen, the Netherlands; 2 Department of Surgery, Division of Abdominal Surgery, University of Groningen, University Medical Center Groningen, Groningen, the Netherlands; 3 Department of Surgery, Martini Hospital, Groningen, the Netherlands; 4 Department of Surgery, Medical Centre Leeuwarden, Leeuwarden, the Netherlands; 5 Department of Surgery, Antonius Hospital, Sneek, the Netherlands; 6 Department of Surgery, Tjongerschans Hospital, Heerenveen, the Netherlands; 7 Department of Surgery, Nij Smellinghe Hospital, Drachten, the Netherlands; 8 Department of Surgery, Division of Pediatric Surgery, University of Groningen, University Medical Center Groningen, Groningen, the Netherlands

**Keywords:** Bowel dysfunction, Colon cancer, Follow-up, Hemicolectomy, Postoperative

## Abstract

Differences in long-term outcomes regarding types of colon resections are inconclusive, precluding patient counseling, effective screening, and personalized treatment.

**OBJECTIVE:**

This study aimed to compare long-term bowel function and quality of life in patients who underwent right or left hemicolectomy or sigmoid colon resection.

**DESIGN:**

This was a multicenter cross-sectional study.

**SETTINGS:**

Seven Dutch hospitals participated in this study.

**PATIENTS:**

This study included patients who underwent right or left hemicolectomy or sigmoid colon resection without construction of a permanent stoma between 2009 and 2015. Patients who were deceased, mentally impaired, or living abroad were excluded. Eligible patients were sent the validated Defecation and Fecal Continence and Short-Form 36 questionnaires.

**MAIN OUTCOME MEASURES:**

Constipation, fecal incontinence (both Rome IV criteria), separate bowel symptoms, and generic quality of life were the main outcomes assessed.

**RESULTS:**

This study included 673 patients who underwent right hemicolectomy, 167 who underwent left hemicolectomy, and 284 who underwent sigmoid colon resection. The median follow-up was 56 months. Sigmoid colon resection increased the likelihood of constipation compared to right and left hemicolectomy (OR, 2.92; 95% CI, 1.80–4.75; *p* < 0.001 and OR, 1.93; 95% CI, 1.12–3.35; *p* = 0.019). Liquid incontinence and fecal urgency increased after right hemicolectomy compared to sigmoid colon resection (OR, 2.15; 95% CI, 1.47–3.16; *p* < 0.001 and OR, 2.01; 95% CI, 1.47–2.74; *p* < 0.001). Scores on quality-of-life domains were found to be significantly lower after right hemicolectomy.

**LIMITATIONS:**

Because of the cross-sectional design, longitudinal data are still lacking.

**CONCLUSIONS:**

Different long-term bowel function problems occur after right or left hemicolectomy or sigmoid colon resection. The latter seems to be associated with more constipation than right or left hemicolectomy. Liquid incontinence and fecal urgency seem to be associated with right hemicolectomy, which may explain the decline in physical and mental generic quality of life of these patients. See **Video Abstract** at http://links.lww.com/DCR/C13.

**DISFUNCIÓN INTESTINAL A LARGO PLAZO Y DISMINUCIÓN DE LA CALIDAD DE VIDA DESPUÉS DE LA CIRUGÍA DE CÁNCER DE COLON: SOLICITUD DE DETECCIÓN Y TRATAMIENTO PERSONALIZADOS:**

**ANTECEDENTES:**

Las diferencias en los resultados a largo plazo con respecto a los tipos de resecciones de colon no son concluyentes, lo que impide el asesoramiento preoperatorio del paciente y la detección eficaz y el tratamiento personalizado de la disfunción intestinal postoperatoria durante el seguimiento.

**OBJETIVO:**

Comparar la función intestinal a largo plazo y la calidad de vida en pacientes sometidos a hemicolectomía derecha o izquierda, o resección de colon sigmoide.

**DISEÑO:**

Estudio transversal multicéntrico.

**AJUSTES:**

Participaron siete hospitales holandeses.

**PACIENTES:**

Se incluyeron pacientes sometidos a hemicolectomía derecha o izquierda, o resección de colon sigmoide sin construcción de estoma permanente entre 2009 y 2015. Se excluyeron pacientes fallecidos, con discapacidad mental o residentes en el extranjero. A los pacientes elegibles se les enviaron los cuestionarios validados de Defecación y Continencia Fecal y Short-Form 36.

**PRINCIPALES MEDIDAS DE RESULTADO:**

Se evaluaron el estreñimiento, la incontinencia fecal (ambos criterios de Roma IV), los síntomas intestinales separados y la calidad de vida genérica.

**RESULTADOS:**

Se incluyeron 673 pacientes con hemicolectomía derecha, 167 con hemicolectomía izquierda y 284 con resección de colon sigmoide. La mediana de seguimiento fue de 56 meses (RIC 41-80). La resección del colon sigmoide aumentó la probabilidad de estreñimiento en comparación con la hemicolectomía derecha e izquierda (OR, 2,92, IC 95%, 1,80–4,75, p < 0,001 y OR 1,93, IC 95%, 1,12–3,35, p = 0,019). La incontinencia de líquidos y la urgencia fecal aumentaron después de la hemicolectomía derecha en comparación con la resección del colon sigmoide (OR, 2,15, IC 95%, 1,47–3,16, p < 0,001 y OR 2,01, IC 95%, 1,47–2,74, p < 0,001). Las puntuaciones en los dominios de calidad de vida fueron significativamente más bajas después de la hemicolectomía derecha.

**LIMITACIONES:**

Debido al diseño transversal, aún faltan datos longitudinales.

**CONCLUSIONES:**

Se producen diferentes problemas de función intestinal a largo plazo después de la hemicolectomía derecha o izquierda, o la resección del colon sigmoide. Este último parece estar asociado con más estreñimiento que la hemicolectomía derecha o izquierda. La incontinencia de líquidos y la urgencia fecal parecen estar asociadas a la hemicolectomía derecha, lo que puede explicar el deterioro de la calidad de vida física y mental en general de estos pacientes. Consulte **Video Resumen** en http://links.lww.com/DCR/C13. *(Traducción—Dr. Yolanda Colorado*)

Worldwide, more than 1 million patients are diagnosed with colonic cancer every year.^1^ Developments in surgical techniques and adjuvant chemotherapy regimens resulted in a current 5-year survival rate of 64%.^2^ This implies that the long-term effects of surgery for colon cancer are becoming important to a growing number of people.

The 3 most performed resections for colon cancer, depending on the location of the tumor, are right hemicolectomy, left hemicolectomy, and sigmoid colon resection. A recent meta-analysis emphasized the magnitude of both constipation-associated and fecal incontinence–associated symptoms after surgery for colon cancer.^3^ More detailed knowledge of the long-term presence of specific bowel symptoms after the 3 different types of colectomies would not only enhance tailored preoperative patient counseling but would also provide the clinician with practical indications for more direct screening and personalized treatment during regular follow-up.

Extreme heterogeneity of the available studies, small cohorts, and the use of many nonvalidated bowel function scores previously precluded comparison of the different types of colon resections.^3^ Such knowledge could possibly improve our understanding of the functioning of different parts of the colon.

The hypothesis was that sigmoid colon resection will lead to more constipation, given previous reports on difficult emptying and/or straining.^4,5^ Patients who underwent right hemicolectomy are expected to experience frequent diarrhea or loose stools,^6^ possibly predisposing them to fecal incontinence. Given the negative influence of constipation and/or fecal incontinence on generic quality of life,^7,8^ there may also be differences in generic quality of life. Therefore, the aims of this study were to determine and compare long-term bowel function and generic quality of life between patients who had undergone right hemicolectomy, left hemicolectomy, or sigmoid colon resection.

## MATERIALS AND METHODS

### Study Design

Between October 2017 and December 2019, this cross-sectional study was performed at 7 Dutch hospitals. The mandatory Dutch ColoRectal Audit registry was searched for patients ≥18 years of age, without a previous colectomy, who had undergone either right hemicolectomy, left hemicolectomy, or sigmoid colon resection for colon cancer with curative intention between 2009 and 2015. Excluded were patients who had either died, were mentally impaired, had a permanent stoma, whose address was unknown, or who lived abroad.

Patients who had signed an informed consent form were invited to complete 2 validated questionnaires: the Defecation and Fecal Continence (DeFeC) questionnaire and the Short-Form 36 (see Supplemental Digital Content 1 at http://links.lww.com/DCR/C8).^9,10^ A link to the digital questionnaires was provided unless the patient preferred to receive a hard copy. The patient data were acquired by 1 investigator who screened all medical records. Adjuvant chemotherapy was administered according to one of the standard regimens: FOLFOX, CAPOX, or capecitabine as a single agent. Radiotherapy in the pelvic region had been administered mainly for previous prostate cancer. The Medical Ethical Review Board of University Medical Center Groningen approved the study (approval code METc 2017/245), and it was performed in accordance with the guidelines on Strengthening the Reporting of Observational Studies in Epidemiology.

### Questionnaires

The DeFeC questionnaire contains questions from widely used scoring systems and criteria for various bowel function problems, including the Rome IV criteria for constipation and fecal incontinence, the symptoms of the low anterior resection syndrome (LARS) score, and the Bristol Stool Scale (Supplemental Digital Content 1 at http://links.lww.com/DCR/C8).^11–13^ The Short-Form 36 is a generic quality-of-life questionnaire containing 36 questions covering 8 domains. The scores range from 0 (bad quality of life) to 100 (good quality of life).^10^

### Definitions

To be diagnosed with constipation according to the Rome IV criteria, patients had to report 2 or more of the following symptoms: straining, lumpy or hard stools, incomplete defecation, anorectal blockage, manual maneuvers to facilitate defecation, and less than 3 spontaneous bowel movements per week.^11^ Additionally, the regular use of laxatives had to be needed to loosen stool. Fecal incontinence was also defined according to the Rome IV criteria and included any involuntary loss of stool at least 2× per month.^12^ Furthermore, different subtypes of fecal incontinence were distinguished: soiling (loss of small amounts of feces), urge incontinence (unable to reach the toilet in time), solid incontinence (loss of solid feces without urge), and liquid incontinence (loss of watery feces). The 5 very disabling bowel symptoms of the LARS score (any flatus or liquid incontinence, altered stool frequency, fecal clustering, and fecal urgency) were also analyzed because of their known negative influence on quality of life.^13^ To define stool consistency, the Bristol Stool Scale was used.

Sigmoid colon resection was defined as the surgical resection of a sigmoid tumor with an anastomosis of >15 cm above the anal verge. Surgical resection of a tumor in the descending colon or distal transverse colon was defined as left hemicolectomy. Follow-up time was defined as the time between completion of the questionnaires and primary surgery or reversal of the temporary stoma. Tumor stage was defined according to the Union for International Cancer Control classification. The Charlson Comorbidity Index was used to score the severity of comorbidities.^14^ The European Perioperative Clinical Outcome definitions were used to specify postoperative complications other than anastomotic leakage or a reoperation.^15^

### Statistical Analysis

Continuous data were reported as means (SDs) or medians (interquartile ranges) and were compared using either ANOVA or the Kruskal-Wallis test. For categorical data, counts and percentages were given and compared using the χ^2^ test. To account for multiple testing, subgroup χ^2^ tests were added. Univariable and multivariable binary logistic regression analyses were performed to identify associations between bowel dysfunction and the 3 types of colon resections. Results were presented as OR with 95% CI. Only relevant univariable variables (*p* < 0.10) or variables with a theoretical confounding effect based on an extensive literature search were included in the multivariable models. Possible interactions were checked. A *p* value of <0.05 was considered statistically significant. Missing data were omitted from statistical analyses. All statistical analyses were performed with IBM SPSS Statistics, version 23.0 (Armonk, NY; IBM Corporation).

## RESULTS

**FIGURE 1.**
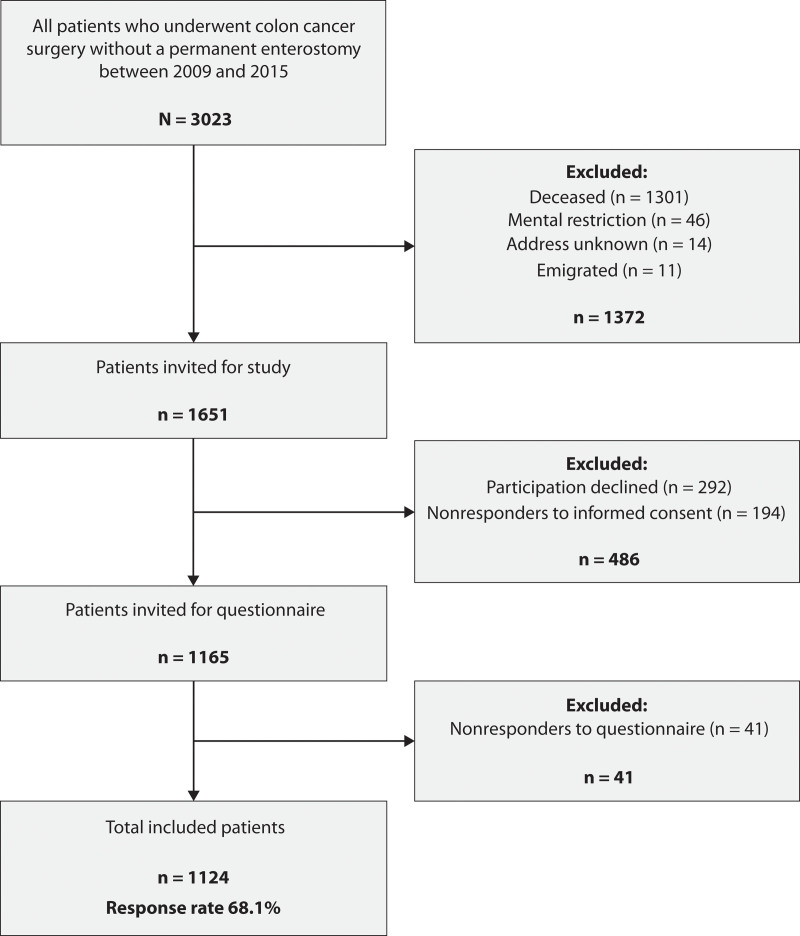
Flow chart of patient inclusion and exclusion.

Between 2009 and 2015, a total of 3023 patients underwent right or left hemicolectomy or sigmoid colon resection for colonic cancer without construction of a permanent stoma. After excluding 1372 patients who had either died, had a mental impairment, or had an unknown or foreign address, questionnaires were sent to 1651 patients, 1124 of whom completed the questionnaires (Fig. 1). Ten of the 1114 included patients (0.9%) had 1 or more missing variable for the definition of constipation or fecal incontinence.

### Patient Characteristics

There were 673 patients who had undergone right hemicolectomy, 167 had undergone left hemicolectomy, and 284 patients had undergone sigmoid colon resection. Taken together, the median follow-up time was 56 (interquartile range, 41–80) months. Patient characteristics according to the type of colon resection are shown in Table 1. A drop-out analysis revealed that more nonresponders had undergone sigmoid colon resections than did responders (31.5% versus 25.3%, *p* = 0.021; see Supplemental Digital Content 2 at http://links.lww.com/DCR/C9). In addition, nonresponders were older than responders and had a higher ASA score (both *p* < 0.001).

**TABLE 1.** Patient characteristics according to the type of colon resection

	Right hemicolectomy, n (%)	Left hemicolectomy, n (%)	Sigmoid colon resection, n (%)	*p* ^a^
Overall, N (%)	673 (100.0)	167 (100.0)	284 (100.0)	
Basic characteristics				
Men	314 (46.7)	99 (59.3)	181 (63.7)	<0.001
Age at surgery (y) ^b^	68.5 (9.7)	65.8 (9.5)	66.2 (9.5)	<0.001**
Follow-up (mo) ^c^	57.0 (42–79)	52.0 (38–79)	58.0 (41–83)	0.121
BMI at surgery (kg/m^2^) ^b^	26.5 (4.2)	26.6 (4.3)	27.6 (4.3)	0.001**
ASA score at surgery				
I	106 (16.2)	34 (21.0)	63 (23.2)	0.016*
II	403 (61.6)	96 (59.3)	172 (63.2)	
III	140 (21.4)	32 (19.8)	34 (12.5)	
IV	5 (0.8)	0 (0.0)	3 (1.1)	
Charlson Comorbidity Index at surgery ^c^	2.0 (2–2)	2.0 (2–2)	2.0 (2–2)	0.218
Previous lower abdominal surgery	239 (35.5)	56 (33.5)	80 (28.2)	0.089
Previous upper abdominal surgery	45 (6.7)	9 (5.4)	20 (7.0)	0.780
Smoking				
No	497 (81.5)	128 (82.6)	211 (83.7)	0.596
Yes	87 (14.3)	19 (12.3)	35 (13.9)	
Recently quit	26 (4.3)	8 (5.2)	6 (2.4)	
Oncologic characteristics				
Tumor stage (UICC)				
I	145 (21.7)	37 (22.2)	102 (36.2)	<0.001**
II	315 (47.2)	65 (38.9)	88 (31.2)	
III	188 (28.1)	61 (36.5)	85 (30.1)	
IV	20 (3.0)	4 (2.4)	7 (2.5)	
Distant metastasis				
No	645 (96.1)	155 (92.8)	264 (93.3)	0.111
Liver	16 (2.4)	6 (3.6)	11 (3.9)	
Lung	5 (0.7)	1 (0.6)	5 (1.8)	
Multiple locations	5 (0.7)	5 (3.0)	3 (1.1)	
Adjuvant treatment				
Previous radiotherapy in pelvic region	14 (2.1)	4 (2.4)	8 (2.8)	0.793
Adjuvant therapy				
No	470 (72.6)	97 (60.2)	196 (71.3)	0.013*
CAPOX	78 (12.1)	38 (23.6)	44 (16.0)	
FOLFOX	63 (9.7)	16 (9.9)	22 (8.4)	
Capecitabine	36 (5.6)	10 (6.2)	12 (4.4)	
Years since last chemotherapy ^b^	4.0 (3.0–6.0)	5.0 (4.0–7.0)	5.0 (3.0–7.0)	0.498
Surgical characteristics				
Setting				
Elective	605 (91.1)	138 (84.1)	248 (89.5)	0.032*
Emergency	59 (8.9)	26 (15.9)	29 (10.5)	
Surgical approach				
Open	391 (58.7)	82 (49.1)	112 (39.6)	<0.001**
Laparoscopic	219 (32.9)	70 (41.9)	145 (51.2)	
Conversion	56 (8.4)	15 (9.0)	26 (9.2)	
Method of anastomosis				
Handsewn	398 (61.9)	107 (71.3)	113 (41.1)	<0.001**
Stapled	245 (38.1)	43 (28.7)	162 (58.9)	
Reconstruction				
Side-to-end	26 (4.0)	17 (10.8)	127 (51.8)	<0.001**
Side-to-side	600 (92.2)	87 (55.4)	59 (24.1)	
End-to-end	25 (3.8)	53 (33.8)	59 (24.1)	
Temporary stoma	15 (2.2)	29 (17.4)	35 (12.3)	<0.001**
Postoperative characteristics				
Anastomotic leakage	24 (3.6)	13 (7.8)	12 (4.2)	0.057
Reoperation	43 (6.4)	16 (9.6)	23 (8.1)	0.305
Other types of complications				
No	446 (66.3)	116 (69.5)	215 (75.7)	0.024*
1 complication	152 (22.6)	40 (24.0)	50 (17.6)	
>1 complication	75 (11.1)	11 (6.6)	19 (6.7)	

CAPOX, Capecitabine and Oxaliplatin; FOLFOX, Folinic acid, Fluorouracil, and Oxaliplatin; IQR = interquartile range; UICC, Union for International Cancer Control.

a *P* value for comparison of the 3 groups.

b Values expressed as medan (SD).

c Values expressed as median (IQR).

* Statistical significance of *p* < 0.05.

** Statistical significance of *p* < 0.005.

### Stool Consistency and Frequency

**FIGURE 2.**
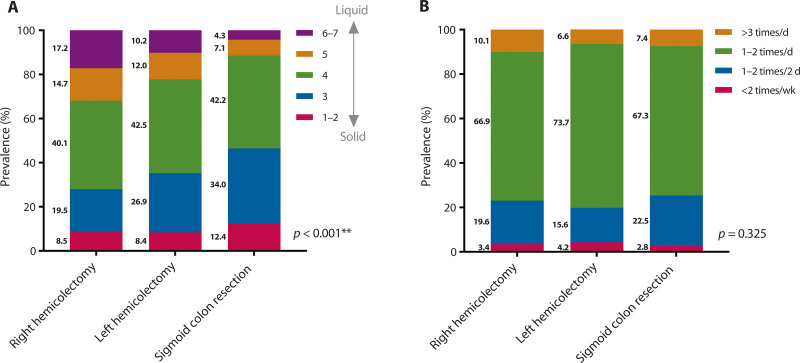
Stool frequency and stool consistency according to the type of colon resection. A, Stool consistency following the Bristol Stool Chart. B, Stool frequency. * Statistical significance of *p* < 0.05. ** Statistical significance of *p* < 0.005.

Figure 2 shows the stool consistency after the 3 types of colon resections. On comparing stool consistency, it was found that the stools of patients who had undergone right hemicolectomy were more liquid compared to patients who had undergone left hemicolectomy and sigmoid colon resection (*p* < 0.001; Fig. 2A). No differences were found for stool frequency (Fig. 2B).

### Individual Bowel Symptoms

**FIGURE 3.**
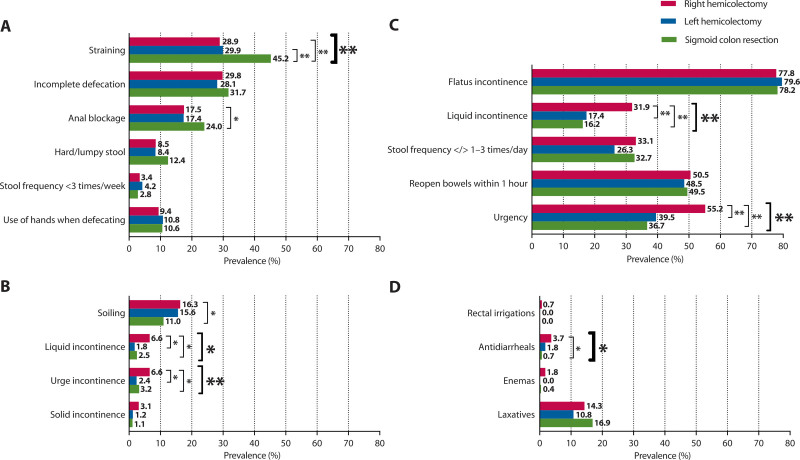
The prevalence of bowel symptoms and defecation treatment according to the type of colon resection. A, Symptoms of constipation. B, Symptoms of fecal incontinence. C, Symptoms of the low anterior resection syndrome score. D, Use of defecation treatment. * Statistical significance of *p* < 0.05. ** Statistical significance of *p* < 0.005.

The prevalence of the different investigated bowel symptoms after the 3 types of colon resections is shown in Figure 3. The prevalence of straining was significantly higher in patients who had undergone sigmoid colon resection compared to patients who had undergone left or right hemicolectomy (45.2% versus 29.9% and 28.9%; *p* < 0.001; Fig. 3A). Regarding fecal incontinence–associated symptoms, liquid incontinence and urge incontinence were both significantly more prevalent after right hemicolectomy than after left hemicolectomy and sigmoid colon resection (6.6% versus 1.8% and 2.5%, *p* = 0.004 and 6.6% versus 2.4% and 3.2%, *p* = 0.021; Fig. 3B). Likewise, liquid incontinence and fecal urgency were the only symptoms of the LARS score that showed a statistically significant difference between patients who had undergone right or left hemicolectomy or sigmoid colon resection (31.9% versus 17.4% and 16.2%, *p* < 0.001 and 55.2% versus 39.5% and 36.7%, *p* < 0.001; Fig. 3C).

### Use of Defecation Treatment

Enemas and laxatives were used to treat constipation in less than 16.9% of the patients without significant differences between the types of colon resections (Fig. 3D). Rectal irrigations and antidiarrheals were used in less than 3.7% of the patients for all types of resections. Antidiarrheals were used most often after right hemicolectomy, compared to left hemicolectomy and sigmoid colon resection (3.7% versus 1.8% and 0.7%, *p* = 0.023).

### Constipation and Fecal Incontinence

Overall, the prevalence of constipation was significantly higher in patients who had undergone sigmoid colon resection compared to patients who had undergone right or left hemicolectomy (31.1% versus 17.7% and 21.0% *p* < 0.001), whereas the prevalence of fecal incontinence was not significantly different (12.7% versus 18.5% and 16.8%; *p* = 0.088).

In accordance with these differences in prevalence, a multivariable model of constipation showed an increased likelihood of constipation in patients who had undergone sigmoid colon resection if right hemicolectomy was taken as the reference category (OR, 2.92; 95% CI, 1.80–4.75; *p* < 0.001). No significant association was found between fecal incontinence and any specific type of colon resection. Additionally, the same univariable and multivariable analyses were performed with left hemicolectomy as the reference category. They also revealed a statistically significant increase in the likelihood of constipation in patients who had undergone sigmoid resection (OR, 1.93; 95% CI, 1.12–3.35; *p* = 0.019; see Supplemental Digital Content 3 at http://links.lww.com/DCR/C10). A direct comparison between patients who had undergone left hemicolectomy versus sigmoid colon resection did not show significant associations for fecal incontinence.

**FIGURE 4.**
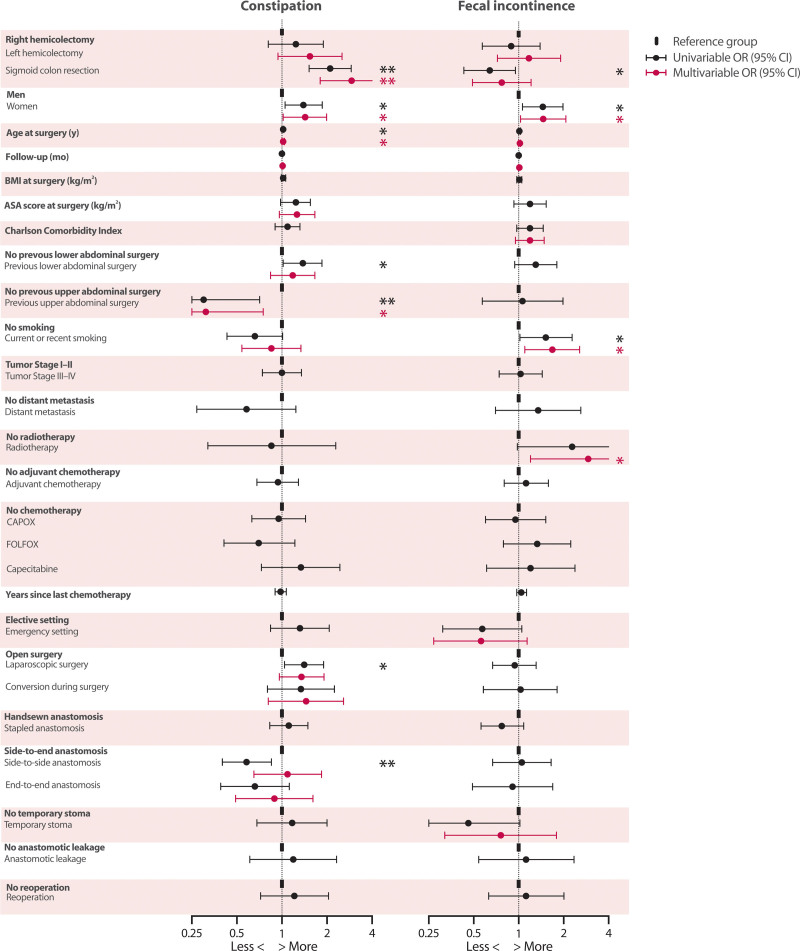
Univariable and multivariable logistic regression analyses of constipation and fecal incontinence. * Statistical significance of *p* < 0.05. ** Statistical significance of *p* < 0.005.

All univariable and multivariable associations between different characteristics and constipation and fecal incontinence are presented in Figure 4. The exact outcomes of these analyses can be found in Supplemental Digital Content 4 at http://links.lww.com/DCR/C11. Women were more likely to experience both constipation and fecal incontinence (constipation: OR, 1.43; 95% CI, 1.02–1.99; *p* = 0.038 and fecal incontinence: OR, 1.46; 95% CI, 1.03-2.07; *p* = 0.036). Likewise, increasing age was also associated with increased odds of constipation (OR, 1.02; 95% CI, 1.00–1.04; *p* = 0.037). Previous upper abdominal surgery was associated with a decreased likelihood of constipation (OR, 0.31; 95% CI, 0.13–0.75; *p* = 0.009). Finally, smoking and radiotherapy were found to be significantly associated with fecal incontinence (OR, 1.68; 95% CI, 1.10–2.56; *p* = 0.017 and OR, 2.92; 95% CI, 1.20–7.08; *p* = 0.018, respectively).

Additionally, using the same multivariable logistic regression model as for overall fecal incontinence, an increased likelihood of liquid incontinence was found in patients who had undergone right hemicolectomy compared to patients who had undergone sigmoid colon resection (OR, 2.15; 95% CI, 1.47–3.16; *p* < 0.001). A similar multivariable logistic regression model of fecal urgency yielded a comparable result (OR, 2.01; 95% CI, 1.47–2.74; *p* < 0.001).

### Generic Quality of Life

**FIGURE 5.**
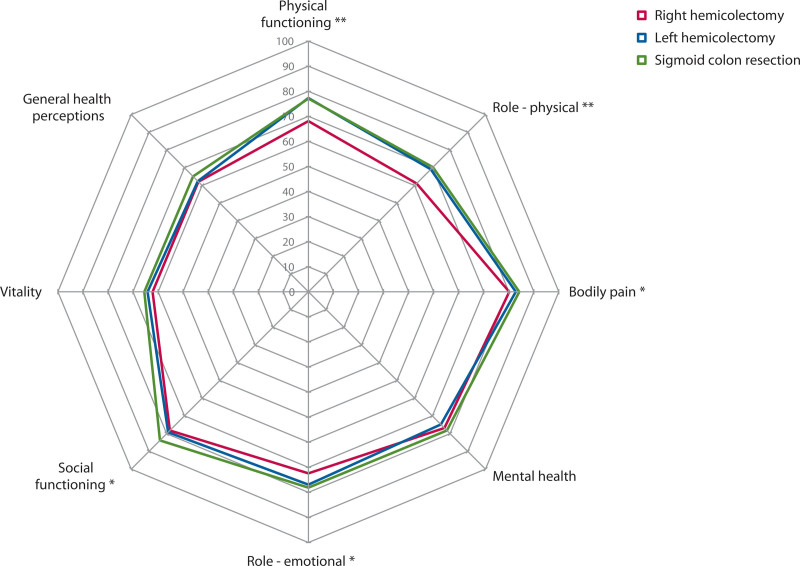
Generic quality-of life scores according to the type of colon resection.

The quality-of-life scores after the 3 different types of colon resections are shown in Figure 5. The scores on different physical domains of quality of life (physical functioning, role-physical, and bodily pain) as well as on different mental domains (social functioning and role-emotional) were significantly lower in patients who had undergone right hemicolectomy (Fig. 5). Subanalysis performed in patients without fecal incontinence still showed worse quality of life on the domains physical functioning and role-physical after right hemicolectomy, whereas no significant difference for the psychosocial domains was observed (see Supplemental Digital Content 5 at http://links.lww.com/DCR/C12).

## DISCUSSION

This study shows that patients who underwent sigmoid colon resection are 3× more likely to experience constipation in the long term than patients who underwent right or left hemicolectomy. This finding corroborates with others who found more constipation-associated problems after sigmoid colon resection compared to patients who underwent a hemicolectomy^16^ or a polypectomy.^17^ Nevertheless, this study is the first to use a validated constipation score to assess patients who underwent resection for colon cancer. Patients who underwent sigmoid colon resection experienced constipation 1.5× more often than the general Dutch population of comparable age (19.8% versus 31.1%).^18^

Various pathophysiological factors have been postulated for the association between sigmoid colon resection and constipation. First, after colonic mobilization, the sensory and motor function of the colon might be reduced because of denervation and fibrosis.^5,16^ Decreased activity of the descending colon and a prolonged transit time were found in patients after surgery for rectal cancer and were attributed to autonomic denervation.^19,20^ These mechanisms are likely to occur after surgery for colon cancer as well. Second, an animal study showed that after 12 week, levels of nitric oxide synthesis increased in rats with a denervated distal colon compared to rats that had not undergone colon surgery.^21^ Nitric oxide might downregulate the contractile activity of the colon and lead to constipation, but this warrants further research.

Right hemicolectomy was associated with twice as much liquid incontinence and fecal urgency compared to sigmoid colon resection. Liquid incontinence was probably linked to the more liquid stool consistency that was found in patients after right hemicolectomy, which had also been reported in previous long-term studies.^22,23^ Comparing stool consistency in patients after right hemicolectomy versus the general population of comparable age illustrates the true increase of liquid to mushy stool after right hemicolectomy (17.2% versus 4.8%, respectively).^24^ Liquid incontinence and fecal urgency were also the 2 symptoms of the LARS score that showed a significantly higher prevalence after right hemicolectomy.

From a pathophysiological point of view, 2 main issues can be distinguished that predispose patients who underwent right hemicolectomy to more liquid stool. First, the absence of the proximal colon, which is known as the part of the colon that absorbs most of the water from the stool.^25^ Second, the absence of or damage to the terminal ileum and/or ileocolic valve may lead to bile acid malabsorption, which causes chronic diarrhea.^6,26,27^ In addition, small-bowel bacterial overgrowth, on account of the absence of the ileocecal valve that acts as a barrier between the flora of the small and large intestine, was proposed as liquifying the stool.^6,27^ Next to that, some hypothesize that injury to the superior mesenteric nerve plexus could result in neurogenic diarrhea,^28^ although more recent studies could not prove this association.^29,30^

Despite the differences in outcomes, personalized treatment after the different types of colon resections seems to be lacking. This may be caused by a lack of awareness among physicians regarding bowel function problems after colon resections. For the rectum resections, postoperative low anterior resection syndrome is receiving more and more attention, but postoperative bowel function problems after colectomies have not been widely investigated. In the current study, only 16.9% of the patients who underwent sigmoid colon resection use laxatives, whereas 31.1% had constipation. Similarly, treatment for fecal incontinence was uncommon. Only 3.7% of the patients who underwent right hemicolectomy were using an antidiarrheal, whereas no less than 6.6% experienced liquid incontinence more than once per month. As bile acid malabsorption is likely to play a role in liquid incontinence after right hemicolectomy, a bile acid sequestrant might relieve these complaints.^27^ However, less than 10% of the patients in this study who had liquid incontinence after right hemicolectomy reported using a bile acid sequestrant, a situation that leaves room for improvement.

All multivariable models were adjusted for sex, age, and follow-up time, as these factors are well known to influence bowel functioning, also in the context of surgery for colon cancer.^5,17,31–33^ The current study shows no effect of follow-up time on constipation and fecal incontinence. This is in contrast with the general consensus that the colon structurally adapts over time after surgery.^6^ These findings imply that prompt treatment of constipation and fecal incontinence is required because the complaints are not likely to resolve spontaneously and might even worsen as more time passes between surgery and follow-up. Concerning smoking, this study shows that smoking seems to be associated with more fecal incontinence after a resection for colon cancer. This might be related to the direct stimulating effect of nicotine on colonic motor activity.^34^ Finally, the current study provides evidence of a 3-fold increase in fecal incontinence in patients who previously received radiotherapy in the pelvic region for other conditions. This emphasizes the detrimental effect of radiotherapy on fecal incontinence, which has been attributed to structural changes in the irradiated tissue.^35^

Remarkably, it seems that adjuvant chemotherapy after surgery for colon cancer does not worsen constipation or fecal incontinence in the long term, which has been noted by others as well.^17,23,32^ However, this is the first study that compares the long-term effects of different chemotherapy regimens and the time since the last chemotherapy treatment, which were both not associated with any of the bowel function problems. Therefore, it seems that the direct cytotoxic effect of chemotherapeutic agents on the mucosa of the GI tract does not have a chronic debilitating impact on patients’ bowel functions, as was suggested previously.^36^ However, future research is required to establish the exact effects of chemotherapy on long-term bowel function.

In line with the findings of more liquid incontinence and fecal urgency after right hemicolectomy, most physical and mental generic quality-of-life domains were worse in these patients. This observation is corroborated by other long-term studies showing impaired quality of life after right hemicolectomy, especially in patients with loose stools.^22,23^ Comparable generic quality of life between patients who had undergone right-sided and left-sided colectomies had been found previously, although shorter questionnaires were used that did not distinguish domains.^37,38^

The large study population, in combination with the validated bowel function scores, strengthens this multicenter study. Although the used scores were not validated for patients who had undergone resection for colon cancer, together, they provide a thorough examination of bowel function that can be compared to other patients and/or to the general population. Furthermore, longitudinal data are lacking. Finally, including long-surviving patients without a permanent stoma, combined with the higher age and worse ASA score of the nonresponders, may have caused selection bias because this would imply that the “most healthy” patients were included in this study. However, this would only reinforce our findings on the large number of bowel function problems after surgery for colon cancer.

## CONCLUSION

This study shows clear differences in long-term bowel function problems after right hemicolectomy, left hemicolectomy, or sigmoid colon resection for colon cancer. Sigmoid colon resection seems to be associated with constipation, with alarmingly low treatment ranges. On the contrary, liquid incontinence and fecal urgency seem to be associated with right hemicolectomy, which may explain the decline in physical and mental generic quality of life of these patients. Hopefully, the current results will provide the clinician with a tool to personalize screening and lead to prompt treatment of both constipation and fecal incontinence during the follow-up of surgery for colon cancer.

## ACKNOWLEDGMENTS

The authors thank I.A.M. ten Vaarwerk and E. Visser for their help with processing the data of the questionnaires. The authors would also like to thank T. van Wulfften Palthe, PhD, for correcting the English article. Finally, they thank all patients for their participation.

## Supplementary Material


