# Supplementary material for: Long-term Bowel Dysfunction and Decline in Quality of Life Following Surgery for Colon Cancer: Call for Personalized Screening and Treatment
Source: Dis Colon Rectum. 2022 Aug 19;65(12):1531–41. doi: 10.1097/DCR.0000000000002377 (PMC9645552; doi:10.1097/DCR.0000000000002377)
Supplement: Supplementary file 3 [file dcr-65-1531-s003.pdf]

Supplemental Digital Content 2. Dropout analysis

	**Responders** No. (%)	**Non-responders** [a] No. (%)	***p* value**
**Overall**	1124 (100.0)	527 (100.0)	
Type of colectomy			
Right hemicolectomy	673 (59.9)	297 (56.4)	*0.021**
Left hemicolectomy	167 (14.9)	64 (12.1)	
Sigmoid colon resection	284 (25.3)	166 (31.5)	
Men	594 (52.8)	235 (44.6)	*0.002***
Age at surgery (years) [b]	67.0 (62 – 75)	72.0 (64 – 78)	*<0.001***
Follow-up (months) [b,c]	55.0 (40 – 78)	61.0 (42 – 80)	*0.079*
ASA score at surgery			
I	203 (18.7)	62 (12.1)	*<0.001***
II	671 (61.7)	279 (54.6)	
III	206 (18.9)	164 (32.1)	
IV	8 (0.7)	6 (1.2)	
Previous abdominal surgery	330 (29.4)	178 (33.8)	*0.070*
Tumor stage (UICC)			
I	284 (25.4)	133 (25.4)	*0.466*
II	468 (41.9)	238 (45.5)	
III	334 (29.9)	139 (26.6)	
IV	31 (2.8)	13 (2.5)	

[a] The clinical data of the non-responders was obtained from the Dutch ColoRectal Audit (DCRA) registry.

[b] Values expressed as median (IQR)

[c] Follow-up since primary rectum or rectosigmoid cancer surgery

* Statistical significance of $p < 0.05$

** Statistical significance of $p < 0.005$

Abbreviations: ASA, American Society of Anesthesiologists; UICC, Union for International Cancer Control.
