# Supplementary material for: Long-term Bowel Dysfunction and Decline in Quality of Life Following Surgery for Colon Cancer: Call for Personalized Screening and Treatment
Source: Dis Colon Rectum. 2022 Aug 19;65(12):1531–41. doi: 10.1097/DCR.0000000000002377 (PMC9645552; doi:10.1097/DCR.0000000000002377)
Supplement: Supplementary file 4 [file dcr-65-1531-s004.pdf]

Supplemental Digital Content 3. Univariable and multivariable logistic regression analysis of constipation and fecal incontinence with left hemicolectomy as the reference category

	Constipation				Fecal incontinence			
	Univariable		Multivariable [a]		Univariable		Multivariable [b]	
Variables	OR (95% CI)	*p* value	OR (95% CI)	*p* value	OR (95% CI)	*p* value	OR (95% CI)	*p* value
**Type of colectomy**								
Left hemicolectomy	Reference		Reference		Reference		Reference	
Sigmoid colon resection	1.70 (1.08–2.67)	*0.021**	1.93 (1.12–3.35)	*0.019**	0.72 (0.42–1.23)	*0.231*	0.67 (0.38–1.20)	*0.177*

[a] The same variables were used as for the multivariable model of constipation with right hemicolectomy as the reference category (see Supplementary Digital Content 3): sex, age at surgery, follow-up, ASA score at surgery, previous lower abdominal surgery, previous upper abdominal surgery, smoking, surgical approach, and type of anastomosis.

[b] The same variables were used as for the multivariable model of fecal incontinence with right hemicolectomy as the reference category (see Supplementary Digital Content 3): sex, age at surgery, follow-up, Charlson comorbidity index at surgery, smoking, radiotherapy, setting, and temporary stoma.

* Statistical significance of $p < 0.05$

** Statistical significance of $p < 0.005$

Abbreviations: CI, confidence interval.
