# Supplementary material for: Long-term Bowel Dysfunction and Decline in Quality of Life Following Surgery for Colon Cancer: Call for Personalized Screening and Treatment
Source: Dis Colon Rectum. 2022 Aug 19;65(12):1531–41. doi: 10.1097/DCR.0000000000002377 (PMC9645552; doi:10.1097/DCR.0000000000002377)
Supplement: Supplementary file 5 [file dcr-65-1531-s005.pdf]

Supplemental Digital Content 4. Univariable and multivariable logistic regression analysis of constipation and fecal incontinence

	**Constipation**				**Fecal incontinence**			
	Univariable		Multivariable		Univariable		Multivariable	
Variables	OR (95% CI)	*p* value	OR (95% CI)	*p* value	OR (95% CI)	*p* value	OR (95% CI)	*p* value
**Type of colectomy**								
Right hemicolectomy	Reference		Reference		Reference		Reference	
Left hemicolectomy	1.24 (0.81–1.89)	*0.326*	1.54 (0.94–2.52)	*0.090*	0.89 (0.57–1.39)	*0.602*	1.17 (0.72–1.91)	*0.532*
Sigmoid colon resection	2.10 (1.52–2.90)	*<0.001***	2.92 (1.80–4.75)	*<0.001***	0.64 (0.43–0.95)	*0.028**	0.77 (0.49–1.21)	*0.259*
**Sex**								
Men	Reference		Reference		Reference		Reference	
Women	1.39 (1.05–1.86)	*0.023**	1.43 (1.02–1.99)	*0.038**	1.45 (1.06–1.98)	*0.021**	1.46 (1.03–2.07)	*0.036**
**Age at surgery (years)**	1.02 (1.00–1.03)	*0.036**	1.02 (1.00–1.04)	*0.037**	1.01 (1.00–1.03)	*0.150*	1.02 (1.00–1.03)	*0.123*
**Follow-up (months)**	1.00 (1.00–1.01)	*0.559*	1.01 (1.00–1.02)	*0.050*	1.00 (1.00–1.01)	*0.348*	1.01 (1.00–1.01)	*0.203*
**BMI at surgery (kg/m^2)**	1.02 (0.99–1.06)	*0.162*	-	-	1.01 (0.97–1.05)	*0.631*	-	-
**ASA score at surgery**	1.24 (0.98–1.55)	*0.068*	1.26 (0.96–1.66)	*0.089*	1.19 (0.93–1.53)	*0.175*	-	-
**Charlson comorbidity index at surgery**	1.09 (0.90–1.32)	*0.396*	-	-	1.19 (0.97–1.46)	*0.088*	1.19 (0.95–1.48)	*0.128*
**Previous lower abdominal surgery**								
No	Reference		Reference		Reference		-	-
Yes	1.38 (1.02–1.85)	*0.035**	1.18 (0.84–1.66)	*0.333*	1.30 (0.94–1.80)	*0.110*		
**Previous upper abdominal surgery**								
No	Reference		Reference		Reference		-	-
Yes	0.30 (0.13–0.71)	*0.006**	0.31 (0.13–0.75)	*0.009**	1.06 (0.57–1.98)	*0.849*		

	Constipation				Fecal incontinence			
	Univariable		Multivariable		Univariable		Multivariable	
Variables	OR (95% CI)	*p* value	OR (95% CI)	*p* value	OR (95% CI)	*p* value	OR (95% CI)	*p* value
**Smoking**								
No	Reference		Reference		Reference		Reference	
Yes or recently quitted	0.66 (0.43–1.01)	*0.057*	0.85 (0.54–1.34)	*0.484*	1.52 (1.02–2.28)	*0.039**	1.68 (1.10–2.56)	*0.017**
**Tumor stage (UICC)**								
I - II	Reference		-	-	Reference		-	-
III - IV	1.00 (0.74–1.35)	*0.992*			1.03 (0.74–1.44)	*0.856*		
**Distant metastasis**								
No	Reference		-	-	Reference		-	-
Yes	0.58 (0.27–1.24)	*0.160*			1.35 (0.70–2.61)	*0.371*		
**Radiotherapy**								
No	Reference		-	-	Reference		Reference	
Yes	0.85 (0.32–2.29)	*0.754*			2.28 (0.98–5.32)	*0.057*	2.92 (1.20–7.08)	*0.018**
**Adjuvant chemotherapy**								
No	Reference		-	-	Reference		-	-
Yes	0.94 (0.68–1.29)	*0.690*			1.12 (0.80–1.58)	*0.509*		
**Type of chemotherapy**								
No	Reference				Reference			
CAPOX	0.95 (0.63–1.44)	*0.803*	-	-	0.95 (0.60–1.52)	*0.829*	-	-
FOLFOX	0.70 (0.41–1.22)	*0.208*			1.33 (0.79–2.23)	*0.280*		
Capecitabine	1.34 (0.73–2.44)	*0.341*			1.20 (0.61–2.38)	*0.601*		
**Years since last chemotherapy**	0.98 (0.90–1.07)	*0.654*	-	-	1.04 (0.97–1.13)	*0.269*	-	-
**Setting**								
Elective	Reference		-	-	Reference		Reference	
Emergency	1.32 (0.84–2.07)	*0.225*			0.57 (0.31–1.05)	*0.073*	0.56 (0.27–1.14)	*0.108*
**Surgical approach**								
Open	Reference		Reference		Reference		-	-
Laparoscopic	1.41 (1.0–1.90)	*0.027**	1.35 (0.96–1.91)	*0.087*	0.94 (0.67–1.31)	*0.698*		
Conversion	1.34 (0.80–2.24)	*0.263*	1.45 (0.81–2.58)	*0.207*	1.03 (0.58–1.81)	*0.922*		

	Constipation				Fecal incontinence			
	Univariable		Multivariable		Univariable		Multivariable	
Variables	OR (95% CI)	*p* value	OR (95% CI)	*p* value	OR (95% CI)	*p* value	OR (95% CI)	*p* value
**Method of anastomosis**								
Handsewn	Reference		-	-	Reference		-	-
Stapled	1.11 (0.83–1.49)	*0.488*			0.77 (0.56–1.08)	*0.127*		
**Type of anastomosis**								
Side-to-end	Reference		Reference		Reference		-	-
Side-to-side	0.58 (0.40–0.85)	*0.005***	1.09 (0.65–1.84)	*0.734*	1.05 (0.67–1.65)	*0.816*		
End-to-end	0.66 (0.39–1.12)	*0.120*	0.89 (0.49–1.61)	*0.697*	0.91 (0.49–1.69)	*0.769*		
**Temporary stoma**								
No	Reference		-	-	Reference		Reference	
Yes	1.17 (0.68–2.00)	*0.571*			0.46 (0.21–1.02)	*0.056*	0.76 (0.32–1.79)	*0.758*
**Anastomotic leakage**								
No	Reference		-	-	Reference		-	-
Yes	1.19 (0.61–2.32)	*0.606*			1.12 (0.54–2.35)	*0.760*		
**Reoperation**								
No	Reference		-	-	Reference		-	-
Yes	1.21 (0.72–2.05)	*0.472*			1.12 (0.63–2.01)	*0.702*		

* Statistical significance of $p < 0.05$

** Statistical significance of $p < 0.005$

Abbreviations: CI, Confidence Interval; ASA, American Society of Anesthesiologists; UICC, Union for International Cancer Control.
