# Supplementary material for: Long-term Bowel Dysfunction and Decline in Quality of Life Following Surgery for Colon Cancer: Call for Personalized Screening and Treatment
Source: Dis Colon Rectum. 2022 Aug 19;65(12):1531–41. doi: 10.1097/DCR.0000000000002377 (PMC9645552; doi:10.1097/DCR.0000000000002377)

**Supplemental Digital Content 5. Generic quality of life scores of all patients without fecal incontinence according to the type of colon resection**

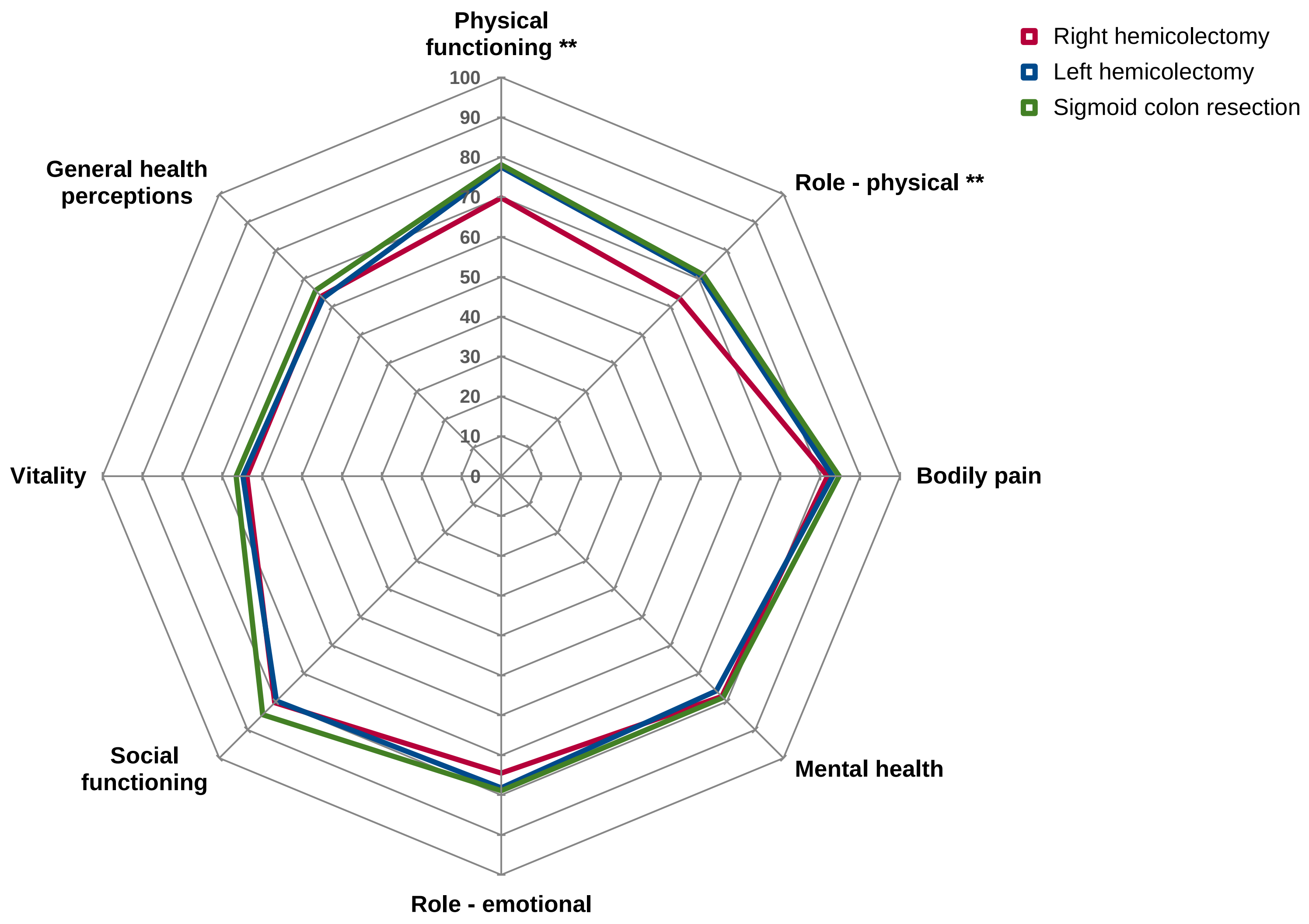

Supplement: Supplementary file 6 [file dcr-65-1531-s006.pdf]
